# Keloids

**Published:** 1873-03

**Authors:** William Augustus Brown

**Affiliations:** Chevalier of the Imperial Ottoman Order of the Medjidieh, Brevet Lieutenant-Colonel United States Volunteers, etc.


### SELECTIONS.

### KELOIDS.

BY WILLIAM AUGUSTUS BROWN, M.D.,

*Chevalier of the Imperial Ottoman Order of the Medjidieh, Brevet Lieutenant-Colonel United States Volunteers, etc.*

Keloid is a name given by Alibert, who originally described the disease in 1810, to a small tumor developed in the subcutaneous areolar tissue, and characterized by red vascular processes, which give an appearance of crabs' claws. The morbid growth which essentially constitutes the keloid of Alibert, takes place in the subcutaneous areolar tissue, between the skin and adipoise membrane, and is an affection which is comparatively rare. It may first appear in the form of one or more small, hard, shining, tubercular-looking elevations, of a roundish or oval shape, attended with itching and pricking pains. The growth of these tumors may proceed for years, and at last attain the size, usually, of an inch or two inches in length and a quarter of an inch in breadth; these dimensions, however, are sometimes very considerably exceeded. The situation is almost always over or near the sternum, and between and upon the mammæ in females. At an uncertain period the tumor becomes somewhat irregular in shape, and displays small, tendinous-looking lines, mingled with small blood vessels of a bluish or purple color, with tapering claw-like processes proceeding from the ends and angles. An objection may be offered to the name, inasmuch as the idea of the crab's claw appearance has given origin to the name of a different and common disease, cancer; and, moreover, keloid disease is not invariably characterized by the vascular processes. Dr. Warren makes three varieties of the disease. 1. A white, permanent elevation of the skin, in the form of the cicatrix of a burn, and sometimes originating in such cicatrix; without discoloration, or very slightly discolored margin; not sensible or painful; differing from the cicatrix of a burn in its disposition to grow, and the difficulty of eradicating it; does not ulcerate or terminate in malignant disease. 2. A

small red pimple, appearing most commonly on the face, radiating red processes into the surrounding skin; size of the body of the tumor usually about one-eighth of an inch, the rays sometimes extending half an inch around it. For a long time after its existence there is no pain or any uneasy sensation. After having continued for years, it assumes a prickling, and finally a burning pain; increases in size, ulcerates, and assumes a cancerous character; never becomes malignant in children or young adults, but in the decline of life, especially in persons who have lived freely. 3. The keloid of Alibert. Like the first, it resembles, in the earlier stages, the cicatrix of a burn. In color it is more red than the surrounding skin, and shoots out red vascular processes, which have the appearance of crabs' claws. The elevation is not great in the common form of the disease; it is sensible to the touch, and attended with a burning, prickling pain. The parts most frequently its seat are the chest, back, shoulders and neck. It may regenerate after removal, and this may happen a number of times. It may eventually ulcerate and become a malignant sore.

On dissection, it exhibits a white fibrinous appearance, much like the schirro-cancerous deposit in the female breast. It is of dense, firm consistence, with little elasticity, and creaks slightly under the knife. It belongs to the class of fibro-plastic growths. Under the microscope, fibres will be observed to cross each other in every possible direction, intercepting spaces occupied by plastic matter of a softer nature. Hardly any vessels will be observed in the interior of the growth.

Dr. Gross, speaking from his large experience, doubts the proneness of the disease to appear upon the chest, and states that it rarely, if ever, degenerates into malignancy; that he never saw a case exhibit such a tendency. He thinks it less rare than generally supposed, and that the division of the disease into several varieties is hardly warrantable; ascribes to it a generally traumatic origin, succeeding upon some local injury, often of a trivial character, the most common exciting causes being scalds and burns. It has been known to appear in a vaccine scar, and the cicatrices left by small-pox, to follow cupping, a blister, and contact with nitric acid. When its origin is spontaneous, it is not always confined to two or three patches, but may appear extensively over the surface of the body, as in a notable case of a negro, described and figured by Dr. Gross, where "it seemed as if the individual was laboring under a keloid diathesis."

Dr. Addison, of London, described before the Medical and Chirurgical Society a form of skin disease which he called *true keloid*, which "originates in the subcutaneous cellular tissue, and is first indicated by a white patch or opacity of the integument, roundish oval shape, scarcely elevated above the surrounding skin, and a more or less vivid zone of redness surrounding the patch. When the patch attained some size, its surface presented a faint yellowish or brownish tint, giving a mottled appearance. With the progress of the disease a hardness and rigidity of the part occurred, accompanied by itching and a sense of pain and constriction, the color changing to reddish, yellowish, or dead-leaf color. The cutis then manifests a disposition to superficial ulceration, and when not excoriated there were often seen tubercular or nodular elevations, the whole resembling the remains of an extensive and imperfectly cicatrized burn. From the boundary of the discolored skin might now be seen tapering, claw-like processes, like the keloid of Alibert." Another case has been published as "true keloid," in which the patient, a young woman, presented a left breast with a diseased surface at the upper part, extending four inches in length by an inch and three-quarters in breadth, presenting a perfectly smooth and polished surface of an opaque, yellow-white color, like polished vellum or ivory, the margin of the diseased portion defined by a strongly marked border of injected vessels, but on the polished surface no vascularity perceptible. No exudation on any part of the breast; nor crust or scurf of any kind. The breast larger than its fellow, and when handled presented several hard, resisting, nodulated tumors. No pain experienced by the patient. Mr. Hodgson was consulted, and pronounced an unfavorable prognosis, as it bore some resemblance to a case which ultimately displayed itself as carcinoma. Sir Benjamin Brodie, who was subsequently consulted in this case, could only adduce a single similar one, in which the process of cure had been effected by throwing off successive layers of diseased skin, during which the extent of surface became continually reduced, the skin beneath ultimately assuming its natural aspect. The treatment was alkaline alteratives, with local application of iodine. In six months the breast had returned to its natural state, the disease, including period of treatment, having continued eighteen months.

The description of these cases might justify the inference that they were nearly allied to lupus. They are certainly quite different from the keloid described by Alibert, and their classification under the head of keloid tends to confusion.

The treatment of the kuloid of Alibert is very unsatisfactory. Dr. Gross states that, so far as he knows, "there is no remedy which will exercise the slightest influence in arresting its development or promoting its removal. Sorbefacients and alterants of every description, and in every form of combination, and in every variety of dose, have been employed, and yet I am not aware of a solitary instance in which their exhibition, however protracted, has been followed by a cure, or even a tendency toward such a result." As to excision, he is decidedly opposed to it. It is only when it interferes with a natural outlet, or becomes a source of great disfigurement, that it should be considered as warrantable. It almost invariably returns after removal, and this disposition seems to be the special characteristic of the disease. "Rising with increased activity, like the phœnix from its ashes, so that the second state is much worse than the first." If ulceration should occur, it should be treated in the ordinary manner, on general principles. There can be little doubt that keloid has sometimes degenerated into malignancy, though this result is so very unfrequent that it may safely be pronounced to be a harmless disease, although troublesome, and frequently attended with considerable pain. In some cases the tumors remain completely stationary for a long time; in others, they gradually advance to a considerable bulk, and then become passive, and so continue for many years. "Occasionally, in consequence, apparently, of prolonged pressure or local irritation, they take on ulcerative action;" and again, on the contrary, the absorbents have been excited by the same means, and their gradual extinction ensue, as in the following cases, which came under my observation:

1. The patient, a gentleman of medium size, nervous temperament, and active habits. In early life, a small, subcutaneous tubercle appeared about midway over the sternum, which continued to enlarge very slowly until it attained the size of a buckshot, when it was removed with a penknife by the patient himself. Immediately the cicatrix manifested evidences of diseased growth; began slowly to enlarge, and became painful—sharp, burning, and lancinating pains, of irregular intensity and duration. During six or eight years, it continued gradually to enlarge until it had attained the size of an almond hull, having given the patient much pain, and been the cause of much mental distress, as he believed it was of malignant character.

He now called a surgeon, who removed the growth by the knife, making two oblong incisions, about half an inch away from the sides of the tumor, which was then raised with a tenaculum, and the flesh removed freely at the base. It was apparently a thorough operation, the morbid growth and a considerable part of the adjacent tissue being removed. As soon as the wound healed, the cicatrix presented the same characteristics, both as to appearance and the darting pains, and the tumor was of twice the former size. Several years afterward, the patient put himself under the care of an irregular practitioner, and submitted to an attempt to remove the disease by means of a caustic application. The escharotic covered a space almost as large as a teacup, and was attended by most intense suffering. The tissues were destroyed to a considerable depth. The sore healed in due time, leaving a large cicatrix, but very soon the disease began to manifest itself in the large scar. Finally, and about twenty years after the first appearance of the little subcutaneous tubercle, the patient went to a hydropathic establishment for dyspepsia, and whilst there, in consequence probably of the frequent and long frictions over the breast with a flesh brush following the baths administered to him, the scar "gathered," as he stated, and discharged about half an ounce of a yellowish ichor, after which it began gradually to pass away, losing its former appearance, and being wholly free from pain. The patient lived several years after this, ultimately dying of phthisis, but never suffered any pain or annoyance afterward from what had been a source of physical and mental suffering for almost twenty-five years.

2. The patient is a brother of the above. When about eighteen years of age, he one day noticed a small pimple on his breast, over the symphisis of the first and second bones of the sternum. It had the appearance of a distended sebaceous follicle, but, upon pressure, yielded no matter. In the course of a month or two he noticed that the pimple had not disappeared, and upon squeezing the skin between his fingers, was sensible of a small tubercle like a bird-shot. No further attention was given to it, but, in the course of a year or more, it was found to have increased to the size of a pea, and began now to be occasionally painful. The growth continued very slowly until, after eight years, it had attained a length of an inch and a half by a quarter of an inch wide, and displayed the characteristic appearance of keloid. He had suffered in the meantime from frequent paroxysms of sharp, lancinating pains, running to the shoulder, and up to the neck. There now appeared another point of disease, about two inches below, which ran through the same course, and presented the same characteristics, as the former. In about sixteen years, the first growth was two and a half inches long, and about half an inch wide; the second, an inch and a half long, and a quarter of an inch wide, both lying transversely across the sternum. The pain was not constant, nor at all times severe, but the paroxysms were frequent, of sharp, cutting pains, darting from the tumors to the neck and left shoulder. Brisk friction with the hand relieved, to some extent, the pain, and the patient states that he would sometimes tear the buttons off his shirt, in his haste to afford himself relief by this means. The want of success which had attended the attempts to cure his brother, and a fear that it might develop a malignant character, and had better be let alone, deterred him from submitting to any kind of treatment. One day, while attending to his affairs, a heavy box fell against him, the edge striking upon these two small tumors on his breast. The pain resulting from this accident is described as being most excruciating, and continuing for some hours. In a few weeks, it was observed that the painful symptoms were less frequent and less severe than formerly, and believing this amelioration was due to the late blow, he adopted the plan of squeezing the keliods between his thumb and finger, increasing the pressure by the use of both hands. This was done generally at night; during the day frequent and brisk friction with the hand. This course was followed up for several months, and both of these troublesome tumors were observed to gradually fade away, and become free from pain, leaving a white scar like an old cicatrix. —*Cincinnati Lancet and Observer.*

